# Long non-coding RNA *FENDRR* inhibits the stemenss of colorectal cancer cells through directly binding to *Sox2* RNA

**DOI:** 10.1080/21655979.2021.1977054

**Published:** 2021-10-26

**Authors:** Xin Zhao, Jincheng Wu, Yongwen Li, Feng Ye, Chunyue Wang

**Affiliations:** a Department of Medical Oncology, The First Affiliated Hospital of Xiamen University, Siming District, Xiamen City, Fujian Province, China; b Department of General Surgery, The First Affiliated Hospital of Xiamen University, Siming District, Xiamen City, Fujian Province, China; c Department of Gastrointestinal Surgery, The First Affiliated Hospital of Xiamen University, Siming District, Xiamen City, Fujian Province, China

**Keywords:** Long non-coding rna, *sox2*, cancer stem cell, mRNA stability, RNA–RNA interaction

## Abstract

Cancer stem cells (CSCs) contribute to malignant features. Long non-coding RNA (LncRNA) *FENDRR* has been shown to regulate tumor proliferation, migration, and invasion. However, the effects of *FENDRR* on the CSC-like traits of colorectal cancer cells remain to be elucidated. Here, we identified that lncRNA *FENDRR* level was remarkably lower in spheres formed by colorectal cancer cells compared to that in parental cancer cells. Further functional experiments showed that *FENDRR* overexpression attenuated the CSC-like traits of colorectal cancer spheres, while *FENDRR* knockdown conferred the CSC-like traits for colorectal cancer cells, as characterized by the alteration of ALDH activity, sphere-formation ability, and expression of stemness markers (*Oct4, Sox2, and KLF4*). RNA–RNA interaction *in vitro* analysis combined with mRNA stability assay revealed that lncRNA *FENDRR* directly interacted with *Sox2* mRNA 3’UTR, reduced its mRNA stability and thus inhibited *Sox2* expression. In addition, lncRNA *FENDRR*-mediated effects on the CSC-like traits of colorectal cancer cells depended on *Sox2* expression. This work suggests that lncRNA *FENDRR* can block the CSC-like traits in colorectal cancer cells through directly interacting with *Sox2* mRNA 3’UTR.

## Introduction

Long non-coding RNA (LncRNA) is a kind of RNAs longer than 200 nucleotides [1]. LncRNA can not or can only encode limited proteins, but it can modulate gene expression at the post-transcriptional or transcriptional level, thus affecting the biological process, for example, lncRNA plays a critical role in transcriptional silencing or activation, nuclear transport, chromosome modification, and so on [2]. In recent years, many abnormal lncRNAs have been found in colorectal cancer and have been regarded as specific biomarkers for prognosis, diagnosis, and even treatment prediction [3]. Additionally, lncRNAs are closely related to the growth and proliferation, invasion and metastasis, apoptosis, and drug resistance of colorectal cancer cells [4,5]. However, the mechanisms contributing to lncRNA-mediated effects on colorectal cancer progression are still confusing.

The theory of cancer stem cells (CSCs) was put forward in 2001 when CSC was regarded to be a critical effector for tumor occurrence, recurrence, and drug resistance [6]. Recently, lncRNAs are also found to be involved in CSC progression or the CSC-like traits of cancer cells, for example, lncRNA *LUCAT1* has been found to be related to the metastasis and TNM staging and promote the self-renewal of breast CSCs [7]; LncRNA *Sox2OT* is shown to promote the CSC-like traits of bladder cancer cells by sponging *miR-200 c* and thus positively regulating *Sox2* expression [8]; And lncRNA *NEAT1* confers cancer stemness and sensitizes cells to chemotherapy in triple-negative breast cancer *(TNBC)* [9]. LncRNA *FENDRR* has been confirmed to be related to tumorigenesis in various tumors, such as migration, invasion, apoptosis, and chemoresistance [10,11]. Notably, it is found that lncRNA *FENDRR* attenuates adriamycin resistance by inhibiting *MDR1* expression [12] and in agreement with this rationale, *FENDRR* reduces the CSC-like traits of non-small cell lung cancer (NSCLC) cells via suppressing *MDR1* expression [13]. However, its effects in colorectal cancer cell stemness remain to be elucidated.

Here, we aimed to explore the effects of lncRNA FENDRR in colorectal cancer stemness. We constructed a colorectal CSC model through collecting spheres by 3D non-adherent culture and found that *FENDRR* was lowly expressed in colorectal cancer sphere. Then, gain- and loss-of functional experiments revealed that *FENDRR* negatively regulated the CSC-like traits of colorectal cancer cells. Mechanistic studies showed that lncRNA *FENDRR* directly interacted with *Sox2* mRNA 3’UTR, but not *Oct4* and *KLF4* mRNA and thus increased *Sox2* mRNA stability and expression. At last, we demonstrated that lncRNA *FENDRR* conferred the CSC-like traits and chemoresistance of colorectal cancer cells dependent on *Sox2* expression.

## Material and methods

### Cell culture

Colorectal cancer cell lines HCT-116 and HT-29 were purchased from Fenghui Biotechnology Co., Ltd (Changsha, China) and cultured in 1640 medium (MeilunBio, Dalian, China) plus 10% FBS (Fetal bovine serum, Oricell, Suzhou, China) as well as 1% penicillin (Sangon, Shanghai, China) and streptomycin (Sangon). Cells were cultured at 37°C in a humidified atmosphere of 5% CO_2_. 5-Fu-resistant HT-29 cells were established by culturing HT-29 cells with 5-Fu (2 μM) for at least three months and then the resistant clones were collected and expanded for a long culture with 100 nM 5-Fu.

### Real-time quantitative PCR (RT-qPCR)

1 mL Trizol reagent (Cat # R401-01, Vazyme, Nanjing, China) was used to extract total RNA of 10^6^ cells. The absorbance value of A_260_ and A_280_ of RNA should be 1.8≤ A_260_/A_280_ ≤ 2.0, and the RNA concentration should be calculated. M-MLV reverse transcriptase (Cat # R021-01, Vazyme) was used to reversely transcribe RNA at 37°C for 1 h in 10 μL system and inactivate the reverse transcriptase at 95°C for 3 min. The synthesized cDNA was saved at −20°C. 20 μL reaction system containing 2 × Quantitative PCR buffer (Cat # Q711-02, Vazyme) 10 μL, 2 μL upstream and downstream primers was used to detect the relative expression levels of transcripts. The reaction conditions were as follows: pre-denaturation at 93°C for 3 min, 93°C for 30 s, 56°C for 40 s. *GAPDH* was served as an endogenous control.

### Western blot

Cells were digested with 0.05% trypsin and 0.02% EDTA for 30 s – 2 min. Then, a complete medium was added to terminate the digestion. The supernatant was centrifuged at 4°C 800 r/min, and added 200 μL 1% SDS protease inhibitor to lyse cells, and the lysate was repeatedly pumped (on ice bath). Protein concentration was quantified following the instructions of Pierce protein assay kit. Before use, freeze-thaw samples on ice, take a certain volume (including 50 μg protein) into a clean Eppendorf tube and add 4 μL 5 × SDS loading buffer, 1% SDS to 20 μL. Denatured at 95°C for 5 min, the samples were placed on ice and loaded as soon as possible. 10% SDS-PAGEs were used to separate the proteins, which were then transferred to the ECL membranes (100 V, 1 h), sealed with 10% no-fat milk for 2 h at 5% PBST, and then incubated with primary antibodies overnight at 4°C. PBST solution was used to wash membranes three times, which were then reacted with horseradish peroxidase labeled Goat anti-mouse Ig at room temperature for 1.5 h. Finally, membranes were exposed using an ECL kit (Cat # E411-03, Vazyme, Nanjing, China) to detect protein expression. GAPDH served as an endogenous control.

### Lentivirus and plasmid construction, infection, and transfection

The *FENDRR* overexpression (FENDRR-oe) and knockdown (FENDRR-kd) lentivirus and control lentivirus vector were purchased from HANBIO (Shanghai, China). The siRNA against *Sox2* (Sox2-kd) and *Sox2* overexpression (Sox2-oe) plasmid were purchased from GenePharma (Shanghai, China). The transfection procedure was performed using jetPRIME (Polyplus, New York, USA) following the manufacturer’s protocols.

### Sphere-formation assay

Sphere formation analysis was performed to evaluate the CSC-like traits of colorectal cancer cells. Briefly, cells were cultured in 37°C, 5% CO_2_ incubator, low-adherent culture plates with sphere-culturing medium containing DMEM/F12 (Cat # 31,331,093, Thermo Fisher Scientific, Waltham, MA, USA) with 1% methylcellulose (Cat # M0512, Sigma) and 10 ng/ml FGF-β (Cat # 11,343,623, ImmunoTools), 10 ng/ml EGF (Cat # 11,343,406, ImmunoTools) and 1 × B27 (Cat # 17,504,044, Thermo Fisher Scientific). Ten days later, sphere size and number were observed under microscope. For experiments of spheres, spheres were collected, re-digested, and subjected to further experiments.

### ALDH activity detection

The Aldehyde Dehydrogenase Activity Colorimetric Assay Kit (Sigma–Aldrich) was used to detect ALDH activity according to the manufacturer’s recommendation.

### Luciferase reporter analysis

The 5’UTR (Untranslated Region), CDS (Coding sequence), and 3’UTR sequences of *Sox2* were cloned into PMIR-Reporter plasmid, referred as PMIR-Sox2-5’UTR, PMIR-Sox2-CDS, and PMIR-Sox2-3’UTR, which were co-transfected into 293 T cells with β-gal plasmid. Then, the luciferase activity and related analysis were referred to the previous study [14].

### *RNA–RNA interaction* in vitro *analysis*

The detailed protocols were referred to the previous study [15]. BrU-labeled RNAs (*FENDRR* and *FENDRR*-Anti-sense) were synthesized from Genepharma (Shanghai, China).

### Analysis on mRNA stability

Colorectal cancer cells were infected with FENDRR-oe and vector, respectively. 48 h later, cells were treated with actinomycin D (Act D) for another 2 h, 4 h, and 6 h, respectively, RNA was extracted and *Sox2* relative expression level was determined by RT-qPCR analysis.

### Cell viability assay

Colorectal cancer cells were seeded into 96-well plates at 4 × 10^3^ cells/well. Cell Counting Kit-8 (CCK-8) kit (GLPBIO, Shanghai, China) was used to measure cell viability. Add 10 μL CCK8 solution to each well on day 1, 2, and 3, respectively. Incubate the plates at 37°C for 2 h. Mix gently on the track vibrator for 1 min to ensure uniform color distribution. Then, the absorbance at 450 nm was measured using a microplate reader to evaluate the cell viability.

### Statistical analysis

Graphpad Prism version 8.0 statistical software was used to analyze the significance between groups. Data were expressed as χ ± s. *T* test was used to compare the two groups. P < 0.05 was statistically significant.

## Results

### *Overexpression of lncRNA* FEDNRR *attenuates the CSC-like traits of colorectal cancer spheres*

**Figure 1.**
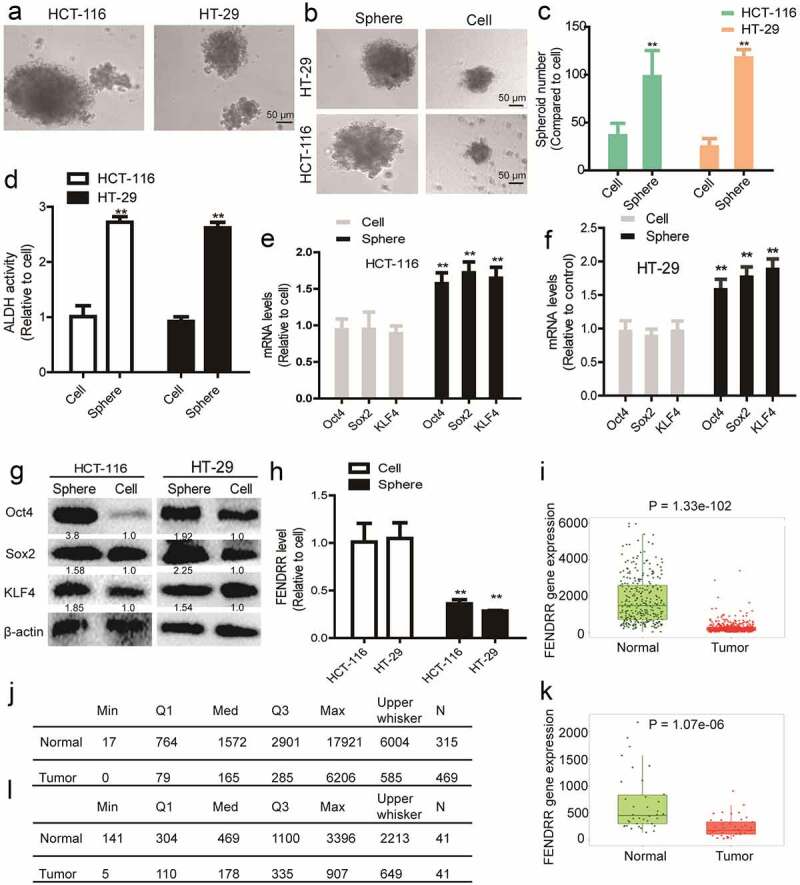
**LncRNA *FEDNRR* level is significantly downregulated in colorectal cancer spheres**. (a) The representative images of spheres formed by colorectal cancer cells. (b) Sphere size was examined in colorectal cancer spheres and cells. (c) Sphere number was measured in colorectal cancer spheres and cells. (d) ALDH activity was evaluated in colorectal cancer spheres and cells. (e and f) The mRNA level of stemness markers (*Sox2, Oct4, KLF4*) was determined in colorectal cancer spheres and cells. (g) The protein level of stemness markers was detected in colorectal cancer spheres and cells. (h) *FENDRR* level was examined in colorectal cancer spheres and cells. (i – l) *FENDRR* expression was detected in data from TCGA using the *Tumor, Normal and Metastatic tissues tool (https://tnmplot.com/analysis/*). n ≥ 3, **P < 0.01 vs. control

**Figure 2.**
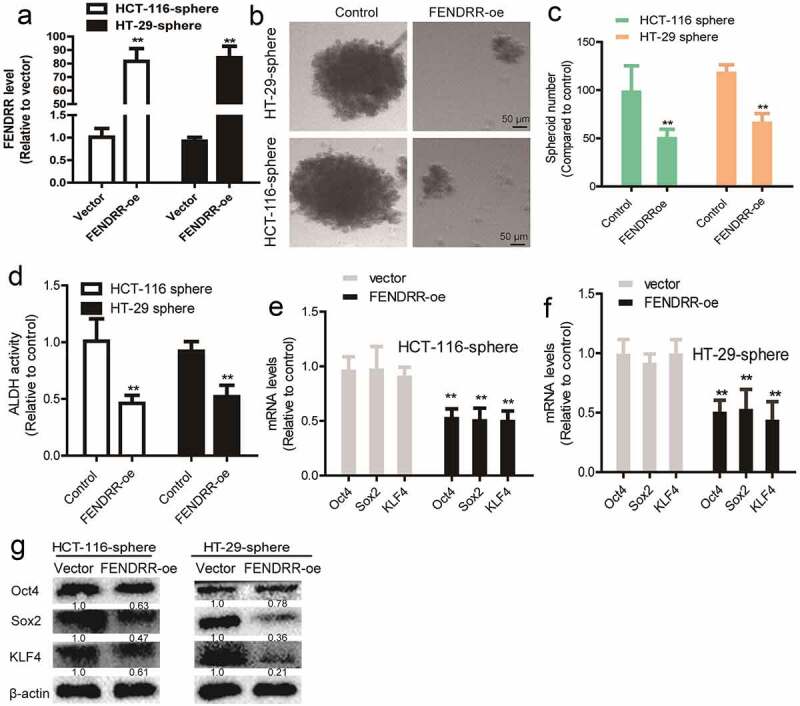
**Overexpression of lncRNA *FEDNRR* attenuates the CSC-like traits of colorectal cancer spheres**. (a) The overexpression efficiency of FENDRR-oe was validated by RT-qPCR. (b and c) Sphere number and size were measured in colorectal cancer spheres with *FENDRR* overexpression or not. (d) Colorectal cancer spheres with or without *FENDRR* overexpression were subjected to ALDH activity detection. (e and f) The mRNA levels of stemness markers (*Sox2, Oct4, KLF4*) were detected in colorectal cancer spheres with or without *FENDRR* overexpression. (g) The protein levels of stemness markers (Sox2, Oct4, KLF4) were detected in colorectal cancer spheres with or without FENDRR overexpression. n ≥ 3, **P < 0.01 vs. control

To investigate the effects of lncRNA *FENDRR* on the CSC-like traits of colorectal cancer cells, the spheres formed by colorectal cancer cells were collected through 3D non-adherent culture which has been confirmed to retain the CSC-like traits in various tumors [16] (Figure 1a). The CSC-like traits were confirmed through examining the sphere-formation capacity (Figure 1b and 1c), ALDH activity (Figure 1d) and expression of stemness markers (Figure 1e – 1g) comparing to the parental colorectal cancer cells. And we identified that lncRNA *FENDRR* level was remarkably lower in colorectal cancer spheres compared to that in the parental cancer cells (Figure 1h). We further evaluated *FENDRR* expression through TCGA data using the *Tumor, Normal and Metastatic tissues tool (https://tnmplot.com/analysis/*) including normal samples from non-cancerous patients and further pediatric tissues, or paired tumor and adjacent normal tissues, and found that FENDRR was indeed lowly expressed in colorectal cancer tissues (Figure 1i – 1l). Then, *FENDRR* was overexpressed in colorectal cancer spheres through lentivirus infection and the overexpression efficiency was validated by RT-qPCR (Figure 2a). It was found that *FENDRR* overexpression attenuated the sphere-formation ability (Figure 2b and 2c), ALDH activity (Figure 2d) and expression of stemness markers (Figure 2e – 2g) in colorectal cancer spheres.

### *Knockdown of lncRNA* FENDRR *confers the CSC-like traits of colorectal cancer cells*

**Figure 3.**
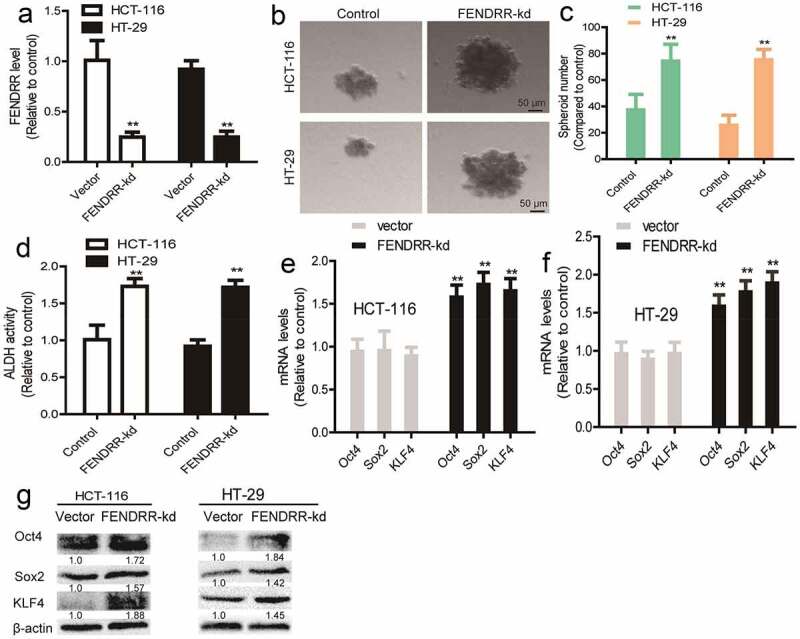
**Knockdown of lncRNA *FENDRR* confers the CSC-like traits of colorectal cancer cells**. (a) The knockdown efficiency of FENDRR-kd was confirmed by RT-qPCR. (b and c) Sphere number and size were determined in colorectal cancer cells with *FENDRR* knockdown or not. (d) ALDH activity was determined in colorectal cancer cells with *FENDRR* knockdown or not. (e and f) The mRNA levels of stemness markers were detected in colorectal cancer cells with *FENDRR* knockdown or not. (g) The protein levels of stemness markers were examined in colorectal cancer cells with or without *FENDRR* knockdown. n ≥ 3, **P < 0.01 vs. control

In contrast, *FENDRR* was knocked down in colorectal cancer cells and knockdown efficiency was validated (Figure 3a). We found that *FENDRR* knockdown enhanced the sphere-formation capacity, as characterized by the increase of sphere number and size (Figure 3b and 3c). In addition, ALDH activity was increased by *FENDRR* knockdown in colorectal cancer cells (Figure 3d). Furthermore, the expression of stemness markers (*Oct4, Sox2, and KLF4*) was increased by *FENDRR* knockdown (Figure 3e – 3g). Thus, these results demonstrate that *FENDRR* can suppress the CSC-like traits of colorectal cancer cells.

### *LncRNA* FENDRR *directly interacts with* Sox2 *mRNA 3’UTR, decreases its stability and expression*

**Figure 4.**
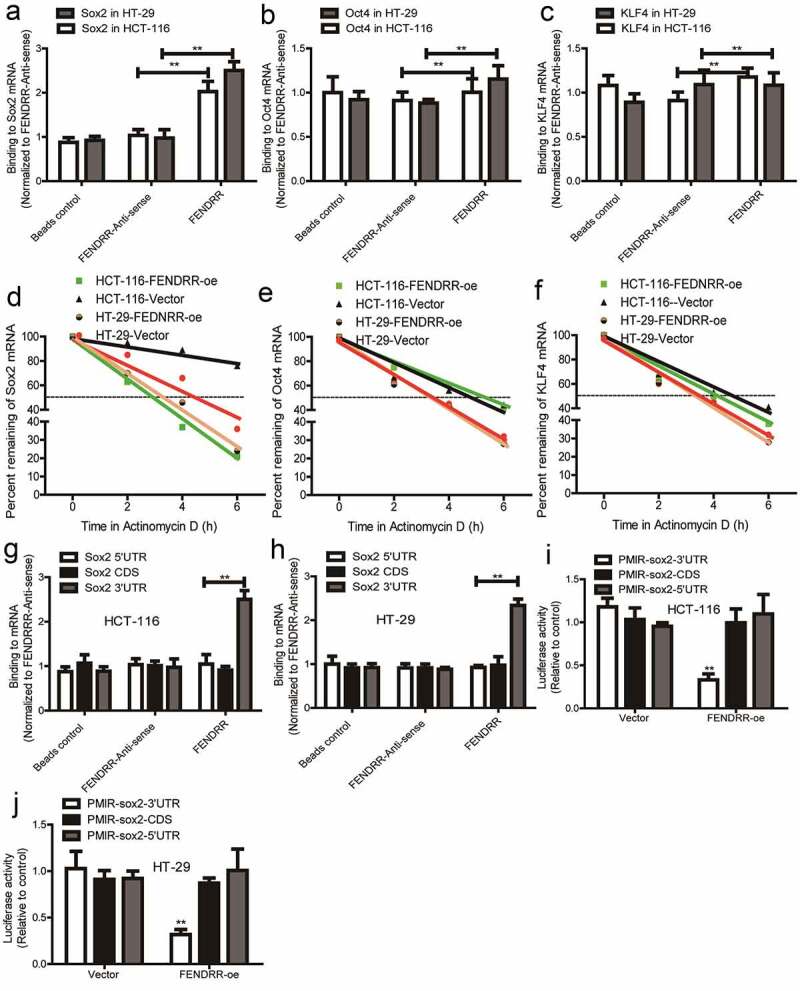
**LncRNA *FENDRR* directly interacts with *Sox2* mRNA 3’UTR, enhances its stability and expression**. (a – c) The interaction between *FENDRR* and *Sox2, Oct4*, or *KLF4* was examined through the RNA–RNA interaction *in vitro* assay. (d – f) The mRNA stability of *Sox2, Oct4* and *KLF4* was determined in colorectal cancer cells with *FENDRR* overexpression or not. (g and h) The interaction between *FENDRR*, and *Sox2* CDS, *Sox2* 5’UTR, or *Sox2* 3’UTR was evaluated in colorectal cancer cells. (i and j) The activity of Luc-Sox2-3’UTR, Luc-Sox2-CDS, and Luc-Sox2-5’UTR was measured in colorectal cancer cells with *FENDRR* overexpression or not. n ≥ 3, **P < 0.01 vs. control

As lncRNAs have been confirmed to act as RNA partners, we wondered whether lncRNA *FENDRR* can act as a partner for the stemness markers (*Oct4, Sox2, and KLF4*). As shown in Figure 4a – 4c, lncRNA FENDRR directly interacted with *Sox2* mRNA, but not *Oct4* and *KLF4* mRNA through RNA–RNA interaction *in vitro* analysis. Consistently, we found that FENDRR overexpression decreased the mRNA stability of *Sox2*, but not *Oct4* and *KLF4* mRNA (Figure 4d – 4f). Additionally, to gain insight about the concrete regions of *Sox2* mRNA bound by *FEDNRR*, RNA–RNA interaction *in vitro* analysis was further performed and we found that *FENDRR* interacted with *Sox2* 3’UTR, but not its 5’UTR and CDS (Figure 4g and 4h). Furthermore, luciferase reporter analysis revealed that *FENDRR* overexpression decreased the luciferase activity of Luc-Sox2-3’UTR, but failed to change the activity of Luc-sox2-CDS and Luc-Sox2-5’UTR (Figure 4i and 4j). Therefore, our results indicate that lncRNA *FENDRR* directly interacts with *Sox2* 3’UTR and thus decreases its mRNA stability and expression.

### *LncRNA* FENDRR *inhibits the CSC-like traits of colorectal cancer cells dependent on* Sox2

**Figure 5.**
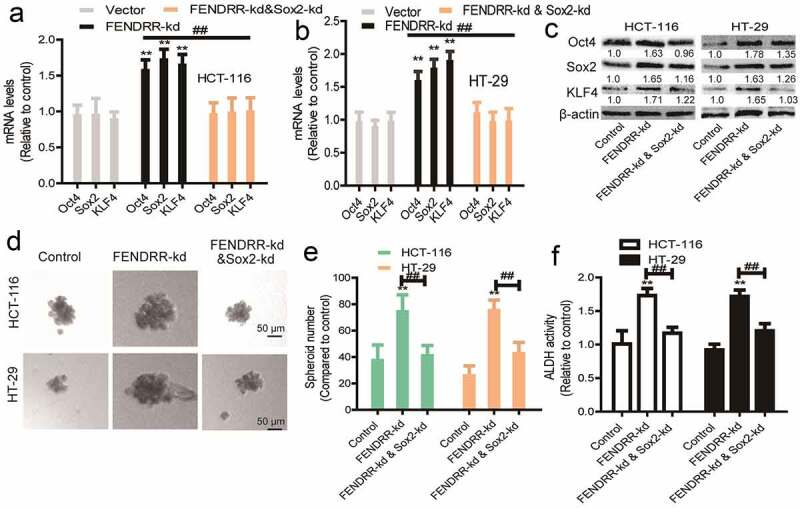
**LncRNA *FENDRR* promotes the CSC-like traits of colorectal cancer cells dependent on *Sox2***. (a and b) The stemness markers’ mRNA levels were examined in cells with *FENDRR* overexpression as well as *Sox2* knockdown or not. (c) The stemness markers’ protein levels were detected in cells with *FENDRR* overexpression as well as *Sox2* knockdown or not. (d and e) Sphere number and size were determined in cells with *FENDRR* overexpression plus *Sox2* knockdown or not. (f) ALDH activity was evaluated in cells with *FENDRR* overexpression as well as *Sox2* knockdown or not. n ≥ 3, **P < 0.01 vs. control, ^##^P < 0.01 vs. FENDRR-oe

We then determined whether *FENDRR* inhibited the CSC-like traits of colorectal cancer cells through *Sox2. Sox2* was knocked down in colorectal cancer cells with *FEDNRR* knockdown. Firstly, it was found that *Sox2* knockdown attenuated the promoting effects of *FENDRR* knockdown on the expression of stemness markers (*KLF4, Oct4*) (Figure 5a – 5c). Secondly, *FENDRR* knockdown-induced enhancement of sphere-formation capacity was partially abrogated by knocking down *Sox2* (Figure 5d and 5e). Thirdly, the increase of ALDH activity mediated by *FENDRR* knockdown was reduced by *Sox2* knockdown (figure 5f). Thus, these results confirm that lncRNA *FENDRR* suppresses the CSC-like traits of colorectal cancer cells dependent on *Sox2* mRNA.

### *LncRNA* FENDRR *inhibits chemoresistance of colorectal cancer cells dependent on* Sox2

**Figure 6.**
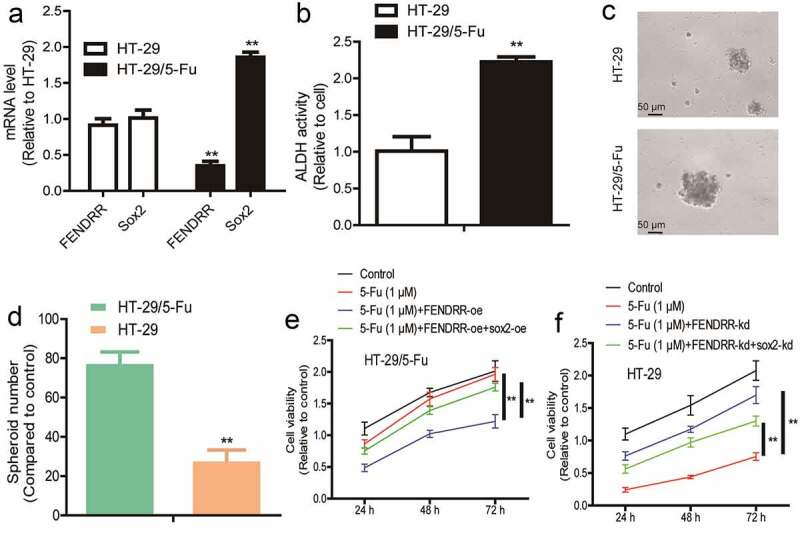
**LncRNA *FENDRR* confers chemoresistance of colorectal cancer cells dependent on *Sox2***. (a) The mRNA levels of *FENDRR* and *Sox2* were detected in HT-29/5-Fu and HT-29 cells. (b) ALDH activity was measured in HT-29/5-Fu and HT-29 cells. (c and d) Sphere number and size were determined in HT-29/5-Fu and HT-29 cells. (e) HT-29/5-Fu cells with *FENDRR* knockdown as well as *Sox2* overexpression or not were subjected to cell viability detection. (f) Cell viability was examined in HT-29 cells with *FENDRR* overexpression plus *Sox2* knockdown or not. n ≥ 3, **P < 0.01 vs. control

Finally, as CSCs can result in drug resistance of tumors, we explored the roles of the *FENDRR/Sox2* axis in chemotherapeutic sensitivity. We initially detected the levels of *FENDRR* and *Sox2* in 5-Fu resistant (HT-29/5-Fu) and sensitive (HT-29) colorectal cancer cells and found that HT-29/5-Fu exhibited a lower level of *FENDRR* and higher level of *Sox2* compared to HT-29 cells, respectively (Figure 6a). Notably, HT-29/5-Fu displayed a stronger CSC-like trait than HT-29 cells, as evident by the increase of ALDH activity, sphere-formation ability (Figure 6b – 6d). Then, *FENDRR* was overexpressed in HT-29/5-Fu cells as well as *Sox2* overexpression or not, it was shown that *FENDRR* overexpression attenuated 5-Fu resistance in HT-29/5-Fu cells, which was rescued by *Sox2* overexpression (Figure 6e). Furthermore, *FENDRR* was knocked down in HT-29 cells as well as *Sox2* knockdown or not, it was identified that *FENDRR* knockdown decreased 5-Fu sensitivity in HT-29 cells, which was partially abrogated by *Sox2* knockdown (figure 6f). Taken together, our results suggest that the *FEDNRR/Sox2* axis suppresses the CSC-like traits and thus drug resistance in colorectal cancer cells.

## Discussion

Here, to explore the roles of lncRNA *FENDRR* in the CSC-like traits of colorectal cancer cells, the spheres formed by colorectal cancer cells through 3D non-adherent culture, which has been shown to enrich CSCs in cancer cells, were collected as a colorectal CSC model [17]. We found that *FENDRR* was lowly expressed in spheres, this promotes us to assume that *FENDRR* can suppress the stemness of colorectal cancer cells. Then, we performed the gain-of functions in spheres and loss-of functions in colorectal cancer cells, respectively. Through analyzing sphere-formation ability, detecting ALDH activity and stemness marker expression, it was found that *FENDRR* negatively regulated the CSC-like traits of colorectal cancer cells. So far, this work, for the first time, revealed the roles of *FENDRR* in the CSC-like traits of colorectal cancer cells.

LncRNAs have been revealed to function through different ways, such as acting as miRNA sponges, RNA or protein partners [1,18]. Additionally, a recent study indicates that lncRNA can encode a small peptide, through which lncRNA suppresses colon cancer progression [19]; A novel primate-specific long non-coding RNA (lncRNA), named FLANC, was identified to promote CRC cell metastasis [20]. And a transcription coactivator, Yes-associated protein 1 (YAP1) – mediated regulation on lncRNA LINC00152 could promote the proliferation and metastasis of CRC cells [21]. Furthermore, a recent work indicated that lncRNA FEZF1-AS1 could facilitate CRC cell proliferation and metastasis by activating STAT3 signaling [22]. In this work, we revealed that lncRNA *FENDRR* directly interacted with *Sox2* mRNA, a critical stemness regulator, and thus decreased *Sox2* mRNA stability and expression, this effect of *FENDRR* on Sox2 mRNA is similar with that of miRNAs on transcripts. Notably, *FENDRR* has been shown to exert similar inhibitory effects on *MDR1* 3’UTR mRNA through competitively binding to *MDR1* mRNA with RNA binding protein HuR [12,13], these results remind us to investigate which areas of *Sox2* mRNA were bound by *FENDRR*. We then performed luciferase reporter assay combined with RNA–RNA interaction *in vitro* analysis, and revealed that lncRNA *FENDRR* directly binds to *Sox2* mRNA 3’UTR. However, it is still unclear whether RNA binding proteins are involved in *FENDRR*-mediated effects on *Sox2* mNRA stability, such as RNA binding protein HuR, this should be explored in the future. In addition, we found that lncRNA *FENDRR* directly interacted with *Sox2* mRNA, not *Oct4* and *KLF4*, the other stemness regulators, and *FENDRR* regulated the expression of *Oct4* and *KLF4* in a *Sox2*-dependent manner, this is consistent with the previous studies showing that *Sox2* is necessary for CDK1- and HIF1α/HIF2α-induced effects on other stemness marker expression [23,24]. Moreover, the previous studies have demonstrated that *FENDRR* is necessary for mammalian embryogenesis [25,26], the procedure of which is similar with that of CSCs, this effect further strengthens the inhibitory effects of *FENDRR* on the CSC-like traits of colorectal cancer cells. Notably, lncRNA *FENDRR* has been shown to repress the protein expression of Sox4, which is another stemness marker and belongs to the same family as Sox2; thus, we wonder whether *FENDRR* can suppress the CSC-like traits of colorectal cancer cells through repressing Sox4 protein, this could be investigated in the future. Furthermore, the effects of FENDRR on the protein stability of Sox2 are still unclear.

## Conclusion

All in all, although *in vivo* experiments are needed, this study reveals a novel *FENDRR/Sox2* axis necessary for the CSC-like traits and chemoresistance of colorectal cancer cells, which might be a novel biomarker for colorectal cancer and chemotherapeutic efficiency.

## Data Availability

All data generated or analyzed during this study are included in this published article.
